# A Novel SND1-BRAF Fusion Confers Resistance to c-Met Inhibitor PF-04217903 in GTL16 Cells though MAPK Activation

**DOI:** 10.1371/journal.pone.0039653

**Published:** 2012-06-22

**Authors:** Nathan V. Lee, Maruja E. Lira, Adam Pavlicek, Jingjing Ye, Dana Buckman, Shubha Bagrodia, Sreesha P. Srinivasa, Yongjun Zhao, Samuel Aparicio, Paul A. Rejto, James G. Christensen, Keith A. Ching

**Affiliations:** 1 Oncology Research Unit, Pfizer Global Research & Development, San Diego, California, United States of America; 2 Global Pre-Clinical Statistics, Pfizer Global Research & Development, San Diego, California, United States of America; 3 Michael Smith Genome Sciences Centre, BC Cancer Research Centre, Vancouver, British Columbia, Canada; 4 Molecular Oncology, BC Cancer Agency, Vancouver, British Columbia, Canada; 5 Department of Pathology, University of British Columbia, Vancouver, British Columbia, Canada; The Moffitt Cancer Center & Research Institute, United States of America

## Abstract

Targeting cancers with amplified or abnormally activated c-Met (hepatocyte growth factor receptor) may have therapeutic benefit based on nonclinical and emerging clinical findings. However, the eventual emergence of drug resistant tumors motivates the pre-emptive identification of potential mechanisms of clinical resistance. We rendered a *MET* amplified gastric cancer cell line, GTL16, resistant to c-Met inhibition with prolonged exposure to a c-Met inhibitor, PF-04217903 (METi). Characterization of surviving cells identified an amplified chromosomal rearrangement between 7q32 and 7q34 which overexpresses a constitutively active SND1-BRAF fusion protein. In the resistant clones, hyperactivation of the downstream MAPK pathway via SND1-BRAF conferred resistance to c-Met receptor tyrosine kinase inhibition. Combination treatment with METi and a RAF inhibitor, PF-04880594 (RAFi) inhibited ERK activation and circumvented resistance to either single agent. Alternatively, treatment with a MEK inhibitor, PD-0325901 (MEKi) alone effectively blocked ERK phosphorylation and inhibited cell growth. Our results suggest that combination of a c-Met tyrosine kinase inhibitor with a BRAF or a MEK inhibitor may be effective in treating resistant tumors that use activated BRAF to escape suppression of c-Met signaling.

## Introduction

Aberrant receptor tyrosine kinase (RTK) activity provides growth and survival signals crucial for the development and progression of many cancers. Treatment of patients with targeted inhibitors of key oncogenic kinase drivers such as sunitinib, erlotinib, gefitinib, and imatinib have demonstrated clinical success [1]. However, despite successful clinical outcomes in select patient populations, the development of resistance to targeted inhibitors can result in disease progression and limit therapeutic effectiveness. Notably, the emergence of secondary mutations or upregulation of compensatory pathways in response to RTK inhibition often arises after a period of initial efficacy [2], [3], [4], [5].

The c-Met/HGFR receptor tyrosine kinase is a promising therapeutic target as mutations of c-Met (in papillary renal cell carcinoma, childhood hepatocellular carcinoma) and focal amplifications of the MET gene locus (in NSCLC, GBM, esophageal and gastric cancers) may indicate an oncogenic dependence on c-Met signaling [6], [7], [8]. For instance, cell lines and xenograft tumors bearing amplification of the *MET* gene locus are very responsive to c-Met inhibitors such as the highly selective small molecule PF-04217903 (METi). Not unlike the eventual resistance that emerges against other RTK inhibitors, several studies have described development of resistance to c-Met inhibitors via c-Met amplification [9] or c-Met mutations that prevent the inhibitor from binding [10]. Additionally, the activation of EGFR/ERBB family receptors [2], [11], [12], KRAS, BRAF, or AKT [13] can also overcome c-Met inhibition.

To anticipate potential resistance, we utilized an *in vitro* screen to select for METi resistant clones of GTL16, a c-Met dependent gastric carcinoma cell line that harbors a high-level focal amplification of the *MET* gene locus [12], [14]. Here we report a novel escape mechanism of GTL16 treated with METi. Molecular characterization of resistant clones reveals a genomic rearrangement resulting in the overexpression of a fusion protein assembled from SND1 and BRAF. SND1 is a multi-functional ribonuclease comprising part of the RNA-induced silencing (RISC) complex [15], [16], [17]. It plays a role in the function of microRNAs (miRNA) and can regulate transcription through transcriptional co-activation, RNA interference, RNA splicing, and RNA editing [18]. Increased expression of SND1 is associated with colon cancer and prostate cancer [15]. Overexpression of SND1 also promotes angiogenesis in hepatocellular carcinoma xenograft models through induction of angiogenic factors [19].

BRAF is a proto-oncogene that promotes cell growth and proliferation by transducing signals from growth factor receptors as part of the MAP kinase pathway via MEK and ERK. Mutations to this protein such as G469A, E586K, V600E, and K601E can increase BRAF catalytic activity [20]. BRAF V600E has been implicated in papillary thyroid carcinoma [21], colorectal carcinoma [22], and melanoma [23]. Similarly, various fusions of BRAF have been implicated in cancer such as pediatric astrocytomas (KIAA1549-BRAF; exons 9/11) [24], melanocytic nevi (FCHSD1-BRAF; exon 9) [25], papillary thyroid carcinomas (AKAP9-BRAF; exon 9) [26], prostate cancer (SLC45A-BRAF; exon 8) [27], and gastric cancer (AGTRAP-BRAF; exon 8) [27].

In our model, the resultant SND1-BRAF fusion protein contains a constitutively active BRAF kinase which increases phosphorylation of ERK. Functionally, this fusion protein signals downstream of c-Met and bypasses its inhibition by METi. We demonstrate that a MEK inhibitor or the combination of c-Met and RAF inhibitors suppresses phosphorylation of ERK and reduces the proliferation of the resistant clones *in vitro.* Together, these findings suggest that targeted inhibitors can be bypassed at multiple levels and that inhibiting the nodes where the signal converges might be a more robust strategy for therapy.

## Materials and Methods

### Generation of Resistant Clones

GTL16 cells were seeded in a 96-well plate at a density of 20,000 cells per well and treated with 0.5 µM PF-0461903 (METi), a selective c-Met kinase inhibitor (Figure S1). The concentration of METi was progressively increased once every two weeks by 0.5 µM increments until a final concentration of 2.5 µM. METi was replenished every 3–5 days as needed. After a total of 4 months, wells with surviving cells at 2.5 µM METi were trypsinized, and subcloned using cloning rings. Three clonal lines, designated GTL16R1, GTL16R3 and GTL16S5 were expanded for further study. The GTL16 gastric cancer cells were a gift from Dr. Paolo Comoglio from the University of Torino Medical School, Candiolo, Italy.

### Cell Viability Assays

Cell viability assays were performed using the GTL16 line and GTL16 resistant clones. Briefly, cells were seeded at a density of 4000 cells/well into 96-well plates and allowed to adhere overnight. The following day, cells were treated with either single agent or combination of METi and a Raf inhibitor, PF-04880594 (RAFi) (Figure S2) or a MEK inhibitor, PD-0325901 (MEKi) [28] as indicated in the figures. For single agent treatment, we administered compound in nine serial concentrations (progressively decreasing from 10 µM to 153 pM by a 4-fold ratio) yielding a full sigmoidal curve. For combinations, we added a second compound (RAFi or MEKi) in five serial concentrations ranging from 10 µM to 39 nM by a 4-fold dilution ratio for RAFi, and from 10 nM to 40 pM by a 4-fold dilution ratio for MEKi. After an additional 3-day incubation at 37°C, 30 µl of Cell Titer Glo (Promega, Madison, WI) was added to indirectly measure cell viability/proliferation using an Envision multi-reader (Perkin-Elmer, Waltham, MA). The BLISS independence algorithm was used to calculate theoretical combination additivity [29]. The ΔBLISS score was calculated as the difference between BLISS and experimentally observed inhibition and ranges from 0 (additive) to 1 (synergistic). Graphical surface plots were rendered using the Lattice R library [30].

### Data Processing

Briefly, readings from the Envision multi-reader were processed using the R package 'drc' (drug response curves) to generate IC50 values [31]. Cell counts were first adjusted by subtracting the average of the baseline cell counts from untreated cells assessed one day after cell seeding. The Tumor Cell Growth Inhibition (TGI) score for each compound concentration was calculated as:



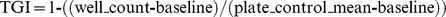
.

The drc package was then used to fit the TGI as a function of the concentration of the compounds. A four-parameter logistic model was used to fit the dose response curves and infer the IC50, slope, and upper and lower limits.

### Analysis of Cell Signaling by Inhibitor Treatment

GTL16 and resistant clones were grown to approximately 80% confluency and then treated with inhibitor compounds or DMSO vehicle control at the indicated concentrations and time duration. For Western immunoblotting, cells were rinsed with phosphate buffered saline (PBS) and subsequently lysed with cell lysis buffer (Cell Signaling, Danvers, MA) supplemented with 2 mM sodium orthovanadate and 2 mM phenylmethylsulfonyl fluoride (PMSF). Cell lysates were harvested, sonicated briefly, and incubated for 1 hr at 4°C. Lysates were then centrifuged at 14,000 rpm for 10 min at 4°C to pellet cell debris, and supernatant was collected.

### Reverse Phase Protein Array (RPPA)

GTL16 and resistant clones were treated with 2.5 µM METi or vehicle control for 1 hr and lysed with CLB lysis buffer according to the vendor’s directions (Zeptosens, Basel, Switzerland). Lysates were sent to Zeptosens for analysis. RPPA data from Zeptosens was processed by the vendor and further processed by median centering. Heatmap plots were generated using a custom R script with scaling to a range of −3 to 3 (arbitrary value).

### Western Immunoblots

The protein concentration of cell lysates was quantified using a DC protein assay kit (Bio-Rad, Hercules, CA). Forty to seventy-six µg of total protein per lane were loaded in a 4–12% gradient BIS-TRIS SDS-PAGE gel (Invitrogen, Carlsbad, CA, NuPAGE) or Criterion XT gel (Bio-Rad), and was transferred onto nitrocellulose membrane (Bio-Rad, #162-0233 or Invitrogen, #IB3010-01). Membranes were blocked with Phosphoblocker (Cell Biolabs, San Diego, CA) and incubated overnight with primary antibody diluted in Phosphoblocker. Primary antibodies were used according to manufacturer’s recommendation: phospho BRAF S445 (Cell Signaling, #2695); BRAF N-term (Cell Signaling, #9433); BRAF c-TERM C-19 (Santa Cruz Biotechnology, Santa Cruz, CA); phospho c-Met Y1349 (Cell Signaling, #3121); c-Met (Cell Signaling, #3127); phospho ERK T202/Y204 (Cell Signaling, #9106); ERK (Cell Signaling, #9102); phospho AKT S473 (Cell Signaling, #4051); AKT (Cell Signaling, #4685); actin (Cell Signaling, #4968).

### Microarrays

RNA and DNA were isolated using RNeasy and DNeasy kits (Qiagen, Germantown, MD) for microarray analysis. Whole genome expression profiling and copy number analysis were performed using Affymetrix HGU133 Plus 2.0 and SNP 6.0 arrays (Affymetrix, Santa Clara, CA), respectively, per manufacturer’s protocol. HGU133 Plus 2.0 data were GC Robust Multiarray Average normalized using R and the gcrma package from bioconductor.org [32].

Affymetrix SNP 6.0 data were processed using the aroma.affymetrix R package according to the methods of H. Bengtsson et al., using the GTL16 as the reference baseline [33].

All array data is MIAME compliant and will be publically available from the Gene Expression Omnibus (GEO) under accession GSE27692.

### 5′ Rapid Amplification of cDNA Ends (RACE)

RNA ligase-mediated, rapid amplification of cDNA ends was performed on total RNA from GTL16, GTL16R1, and GTL16R3 clones using a Generacer™ kit (Invitrogen, Carlsbad, CA). PCR primers specific for 5′ generacer oligo sequence [5′-cgactggagcacgaggacactga -3′] and 3′ primer targeting *BRAF* exons 11 and 12 junction [5′-catcaccatgccactttcccttgt-3′] were used to amplify a first-strand cDNA generated using random hexamers. PCR was performed using 1X Titanium Taq PCR buffer, 0.2 mM dNTP, 0.4 µM forward generacer and reverse BRAF primers, and 1X Titanium Taq DNA polymerase (Clontech Laboratories, Mountain View, CA). The sequence of cycling was 1 cycle at 95°C for 60 sec; 5 cycles at 95°C for 15 sec, 72°C for 90 sec; 5 cycles at 95°C for 15 sec, 70°C for 90 sec; 25 cycles at 95°C for 15 sec, 68°C for 90 sec, and an additional extension at 68°C for 7 min using Peltier Thermal Cycler 200 (MJ Research, Waltham, MA). The PCR product was run on a 1.2% agarose gel and a 2.1 Kb amplicon was gel-purified, cloned into pCR®4-TOPO®R cloning vector (Invitrogen, Carlsbad, CA), and sequenced using M13 forward and reverse primers on a 3730XL capillary sequencer (Applied Biosystems, Foster City, CA). Sequence data was analyzed using Sequencher (Gene Codes Corporation, Ann Arbor, MI).

### Kinase Selectivity Screens

The METi and RAFi were screened against kinase panels (SelectScreen, Invitrogen; University of Dundee, Division of Signal Transduction Therapy, UK; Upstate Biotechnology/Millipore, Billerica, MA). In these selectivity assays the concentration of ATP was set to the Km value of ATP for each kinase tested to allow for a relative potency comparison between the various kinases. The compounds were tested at 1 µM final concentration. Some of the kinases that showed modest potency in the percent inhibition biochemical assays were further evaluated in a dose response cell based assay. For example METi showed biochemical inhibition of IGF1R but did not inhibit IGF1R kinase activity in the follow-up cell based assay.

### Next Generation Sequencing

RNA-Seq libraries were generated and quality assessed as previously described [34], [35] and sequenced on Illumina GA2 instruments. Paired end reads (75 bases each) were aligned to RefSeq transcripts using the BWA alignment tool [36] on default settings and processed to BAM files with samtools [37]. BAM files were deposited into the European Nucleotide Archive under accession ERP001432. Aberrantly mapped pairs were identified using the BAM_preprocessingPairsMisMatch.pl script from SVDetect (r0.6d) [38]. Frequency of mismatched transcripts was counted using a custom script. All read pairs for the potential fusion transcript were extracted with samtools from the original BAM file and *de novo* assembled into contigs using velvet (v1.0.13) [39] and further constructed into transcripts using oases (v0.1.16) (http://www.ebi.ac.uk/~zerbino/oases/). Putative fusion transcripts were then aligned by BLAST [40] to the original RefSeq transcripts (SND1 NM_014390.2 and BRAF NM_004333.4) to identify the composition of the transcript and the position of the fusion junction. The sequence for the most complete transcript was deposited in GenBank under accession JX013981. Coverage data was generated using the pileup function from samtools.

### Fusion Search in Public CNV Datasets

Computational search was performed using publically available CNV (Copy Number Variation) arrays from:

TCGA (http://tcga-data.nci.nih.gov/tcga/tcgaHome2.jsp).

Sanger (http://www.sanger.ac.uk/genetics/CGP/Archive/).

Broad (http://www.broadinstitute.org/tumorscape/pages/portalHome.jsf).

GEO (http://www.ncbi.nlm.nih.gov/geo/) (GSE13429, GSE14437, GSE14960, GSE14994, GSE15096, GSE15264, GSE15526, GSE15688, GSE16619, GSE17247, GSE17958, GSE18333, GSE18828, GSE19416, GSE19539, GSE20709, GSE21780, GSE21990, GSE22208, GSE22306, GSE22615, GSE23300, GSE23452, GSE25016, GSE25839, GSE27560, GSE7822, GSE9829).

GSK (https://cabig.nci.nih.gov/caArray_GSKdata/).

## Results

### Generation and Characterization of METi Resistant GTL16 Clones

**Figure 1:**
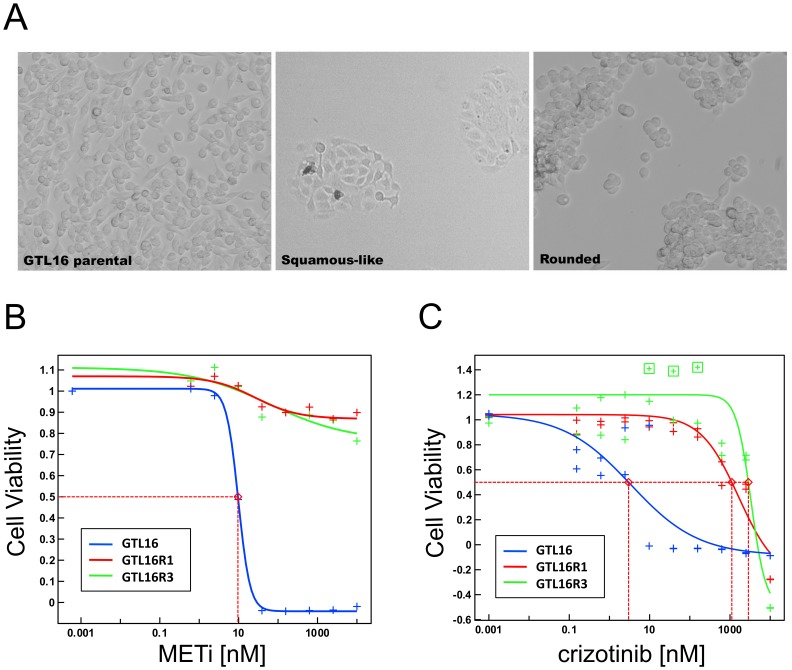
The GTL16 gastric carcinoma cell line becomes resistant to c-Met inhibition after 4 months of continuous treatment with METi. (A) METi-resistant cells exhibit distinct squamous-like and rounded morphologies compared to GTL16 cells. 100X magnification. (B) Cell viability response curves to METi. METi inhibits GTL16 (blue) with an IC50 value of 10 nM, while it does not inhibit cell viability in clones GTL16R1 and GTL16R3 (red and green). Inhibition of cell viability is normalized relative to untreated control cells. (C) Cell viability response curves to crizotinib. Crizotinib inhibits GTL16 (blue) with an IC50 value of 3 nM. While IC50 values for GTL16R1 and GTL16R3 are >1 µM.

GTL16 cells were cultured in the presence of increasing concentrations of METi up to 2.5 µM over a period of four months. Following treatment, GTL16 cells that grew at a rate similar to untreated GTL16 cells were defined to be resistant. The resulting cells from distinct wells appeared as a homogenous population that appeared either rounded (R) or squamous-like (S) (Figure 1A). Resistant clones, GTL16R1 and GTL16R3 did not respond to growth inhibition by METi at concentrations up to 10 µM, while GTL16 were sensitive with an IC50 of 10 nM (Figure 1B). To determine if resistance was a compound specific or a target specific phenomena, we also treated the GTL16R1 and GTL16R3 clones with the c-Met/ALK inhibitor, crizotinib. We observed an IC50 value >1 µM for the resistant clones, while GTL16 had an IC50 of 3 nM (Figure 1C). Data for the rounded clones (GTL16R1 and GTL16R3) are presented below. The squamous clones are pending further characterization but are resistant to c-Met inhibition by a different but unknown mechanism that does not involve BRAF or the c-Met receptor directly.

### Molecular Profiling to Understand Resistance Mechanisms

**Figure 2:**
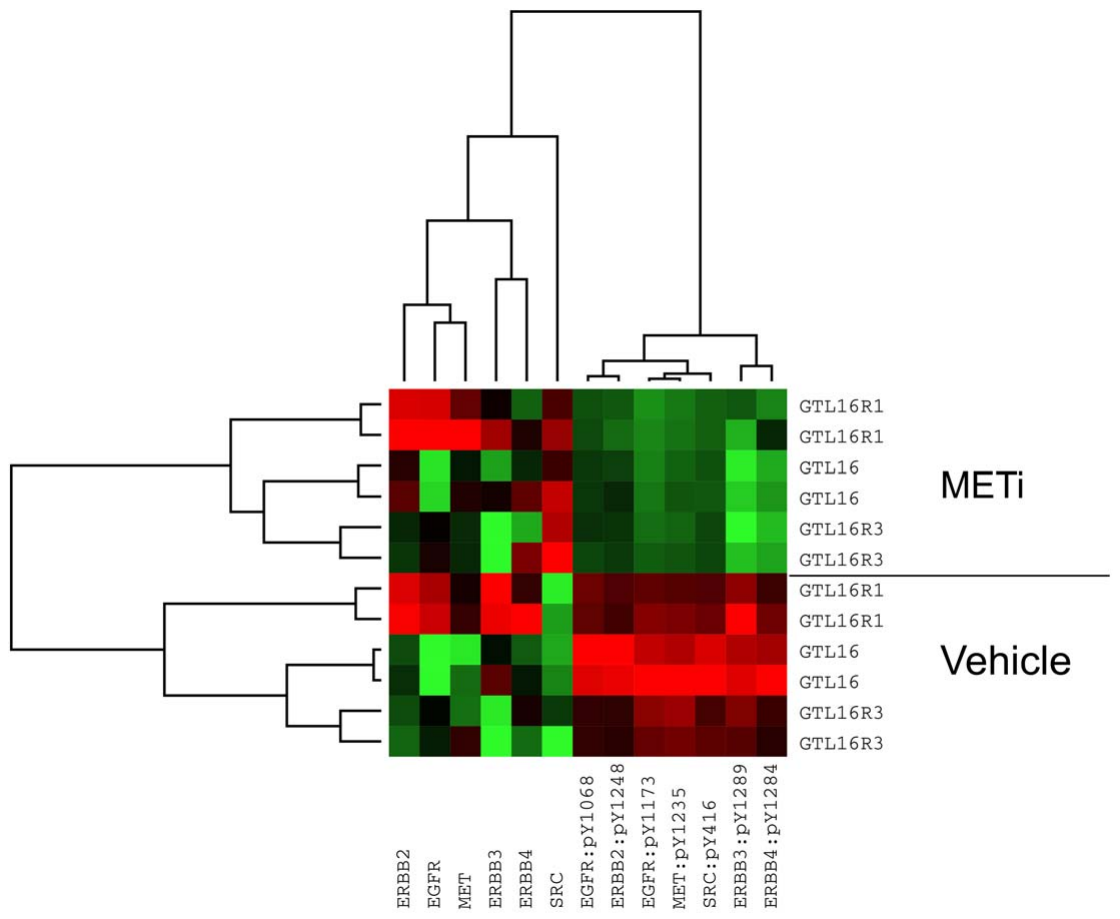
METi inhibits c-Met phosphorylation in both GTL16 and resistant GTL16R1 and GTL16R3 clones. Heatmap of Reverse Phase Protein Array (RPPA) showing GTL16 and resistant clones GTL16R1 and GTL16R3 relative level of total and phosphorylated proteins. Red indicates higher intensity vs green which indicates lower intensity. Cells were treated with DMSO vehicle or METi (2.5 µM) for 1 hr.

To examine the effect of METi on the activation state of signal transduction pathways, protein lysates from GTL16 and the resistant clones were assayed with Reverse Phase Protein Arrays (RPPA) for select total and phosphorylated proteins. Treatment of GTL16, GTL16R1, and GTL16R3 with 2.5 µM METi for 1 hr inhibited phosphorylation of c-Met residue Y1235 (Figure 2). Total c-Met protein levels were slightly elevated in the METi-treated samples compared to vehicle-treated samples, showing that the decrease in phosphorylation was not simply due to a reduction in the amount of total protein (Figure 2). Inhibition of the Y1235 autophosphorylation site, which is critical for c-Met receptor kinase activation [41], indicates that the mechanism of resistance in clones GTL16R1 and GTL16R3 is not due to an inability of METi to bind c-Met kinase and inhibit its catalytic activity. Furthermore, a compensatory increase in c-Met expression in the GTL16R1 and GTL16R3 clones is insufficient to sequester the compound and restore signaling. Interestingly, tyrosine phosphorylation of EGFR, ERBB2, ERBB3, ERBB4, and SRC was also inhibited upon METi treatment. One explanation for this decrease is that overexpressed c-Met transactivates these proteins (Figure 2). The relative fold change of phosphorylation by METi treatment is summarized in Figure S3.

**Figure 3:**
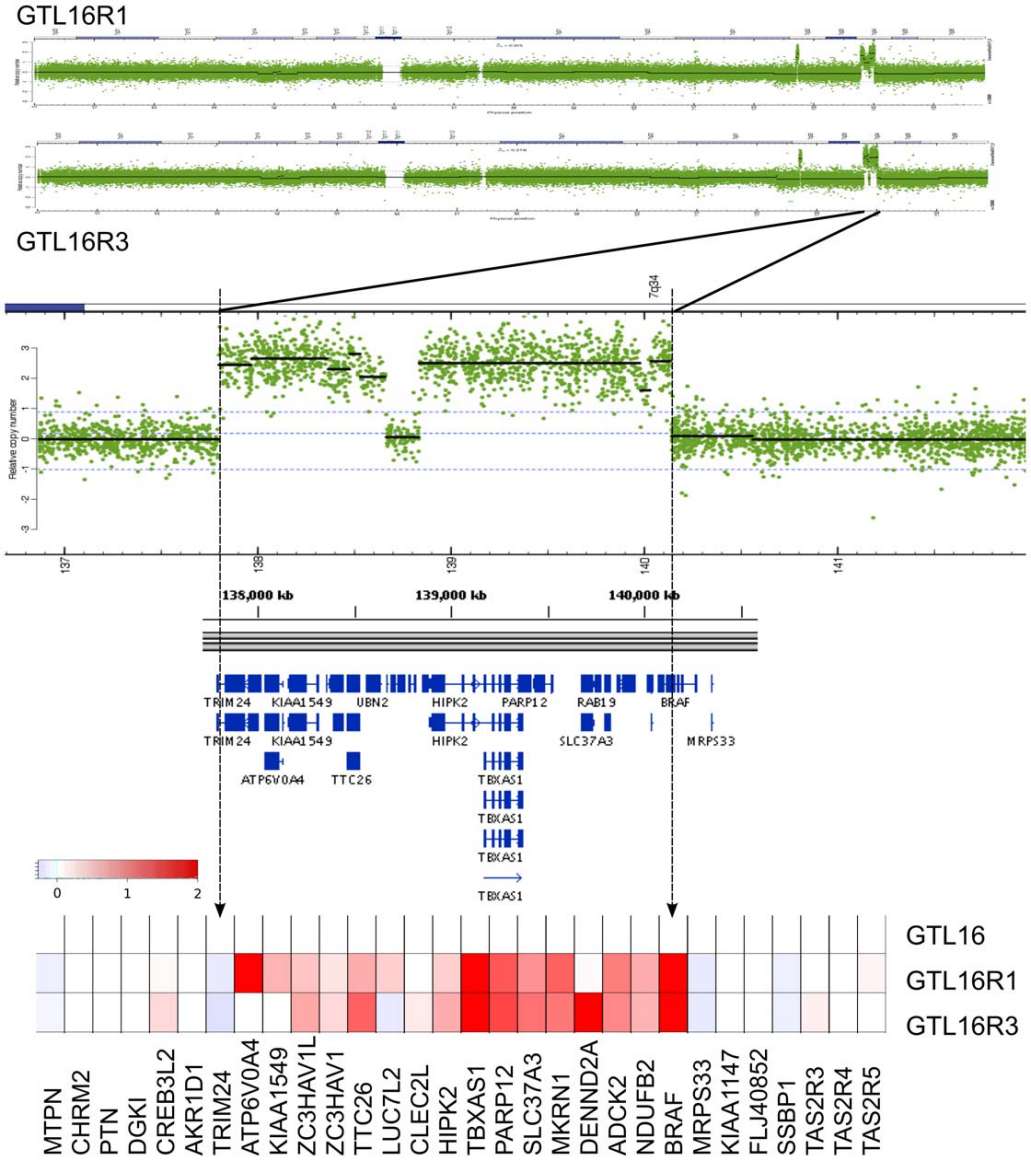
Copy Number Variation (CNV) indicates that BRAF is amplified and bisected in resistant clones. Relative to GTL16, CNV analysis identifies a region of increased copy number at 7q34, with a breakpoint within the *BRAF* locus in GTL16R1 and GTL16R3. In conjunction with Affymetrix expression analysis, genes within the amplified regions are coordinately over-expressed (indicated in red) normalized to GTL16. Expression of the BRAF transcript is increased over GTL16.

To further assess the mechanism of resistance to c-Met inhibition, METi-resistant clones were profiled for gene expression with Affymetrix U133 Plus 2.0 chips and for DNA Copy Number Variation (CNV) using Affymetrix SNP 6.0 arrays. GTL16R1 and GTL16R3 clones have a nearly identical CNV profile to GTL16 except for a 4-fold amplification of ∼356 KB in 7q31 and ∼2 megabases within 7q34. Relative to GTL16, most genes within 7q34 had a corresponding increase in mRNA expression with BRAF expressed more than 32-fold in both GTL16R1 and GTL16R3 (Figure 3).

### BRAF Fusion Identification

**Figure 4:**
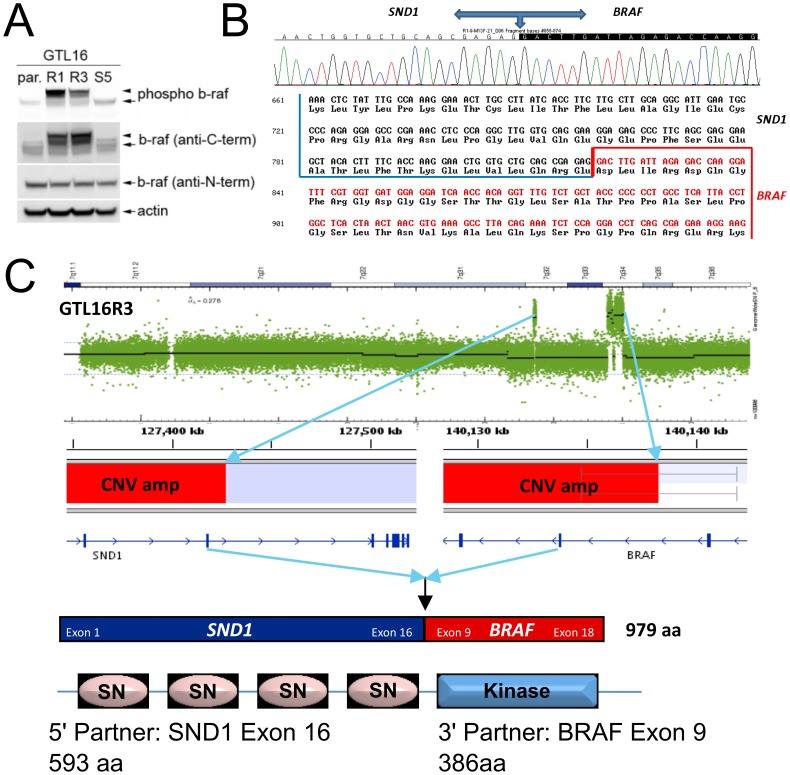
Chromosomal rearrangement at 7q32 and 7q34 results in a highly expressed SND1-BRAF fusion protein. (A) Western Immunoblot of total protein lysates identify a higher molecular weight band (arrowhead) recognized only by the anti-C-terminal BRAF antibody, and present exclusively in GTL16R1 and GTL16R3, consistent with a fusion event within BRAF. Additionally, the putative fusion BRAF is highly expressed and hyperphosphorylated compared to wild-type BRAF (arrow in BRAF panels). (B) 5′ RACE identified the nucleotide sequence of fusion junction spanning exon 16 of SND1 and exon 9 of BRAF. (C) Schematic representation of CNV amplification of the N-terminal portion of SND1 (mapping from exon 1 to 16) and the C-terminal protein of BRAF (mapping from exon 9 to exon 18).

Affymetrix SNP 6.0 array analysis indicated a segmentation breakpoint that bisected the genomic BRAF gene structure within intron 8 (Figure 3). BRAF fusions have been reported previously, and these commonly occur at exon 9 with an intact BRAF catalytic region [24], [25], [26], [27], [42]. We probed GTL16, GTL16R1, GTL16R3, and the squamous-like (GTL16S5) resistant clone lysates by Western immunoblot with N-terminal-, C-terminal-, and phospho-specific BRAF antibodies. All three BRAF antibodies identified wild-type BRAF in all samples (Figure 4A, arrows). By contrast, the C-terminal and phospho-specific BRAF antibodies identified a higher molecular weight species in clones GTL16R1 and GTL16R3 that was absent in GTL16 and GTL16S5 (Figure 4A, arrowheads). The N-terminal-specific BRAF antibody was not able to recognize the higher molecular weight species, suggesting that both the GTL16R1 and GTL16R3 clones express a species of BRAF lacking the N-terminal domain. Interestingly, the higher molecular weight BRAF variant appears to be highly phosphorylated at S445 compared to wildtype BRAF. Based on these observations, we suspected a putative BRAF fusion protein and sought to identify the N-terminal fusion partner. Using 5′ RACE of GTL16R1 and GTL16R3 transcripts with a 3′ primer specific for BRAF exons 11 and 12 produced a 2.1 kb product. Sequencing of the amplicon revealed *SND1* (staphylococcal nuclease and tudor domain containing 1) to be the fusion partner (Figure 4B). *SND1* is normally located on chromosome 7q31 approximately 12 Mb upstream of *BRAF* (7q34). Here, SND1 happens to be bisected by the small 7q31 amplification observed in the GTL16R1 and GTL16R3 clones. The translocation yields a *SND1*-*BRAF* fusion transcript with exon 16 of *SND1* fused in-frame to exon 9 of *BRAF*, resulting in a fusion transcript coding for SND1 amino acids 1-593 coupled to BRAF amino acids 381–766 (Figure 4B and C). Global copy number analysis confirms that the exons adjacent to the amplification breakpoints of SND1 and BRAF are consistent with this junction (Figure S4).

### Confirmation of SND1-BRAF Fusion Sequence

Using Illumina sequencing with GTL16, GTL16R1, and GTL16R3 transcripts, we confirmed that the fusion junction was identical to the sequence obtained by 5′ RACE, and no other nucleotide changes were observed for SND1 or BRAF. *De novo* assembly of read pairs mapping to either SND1 or BRAF produced a fusion transcript that consisted of nucleotides 1-2006 of RefSeq transcript NM_014390.2 (SND1) and 1202–2947 from RefSeq transcript NM_004333.4 (BRAF). Wild type BRAF transcripts were observed in the GTL16R1 and GTL16R3 clones as evidenced by paired reads spanning between exons 8 and 9 (data not shown). GTL16 did not contain any paired reads where one end mapped to SND1 and the other mapped to BRAF. In contrast, GTL16R1 contained 628 chimeric pairs and GTL16R3 contained 702 chimeric pairs (Figure S4).

Consistent with a fusion transcript of SND1 and BRAF, we observed uneven coverage distribution of the N-terminal region of SND1 and the C-terminal region of BRAF. We computed normalized reads per base per million reads (RBM) to compare transcript coverage between samples. Relative to GTL16, the RBM of bases 1-2006 of SND1 in GTL16R1 and GTL16R3 is approximately 2-fold higher, while bases 2007–3522 are equivalent (Figure S5). Similarly for BRAF, bases 1-1201 have about the same RBM in GTL16, GTL16R1 and GTL16R3, while bases 1202–2947 have about 20-fold more RBM in GTL16R1 and GTL16R3 compared to GTL16. There is a steep change in coverage at the fusion junction point at position 2006 for SND1 and 1202 for BRAF. (Figure S5).

### Determination of the Functional Relevance of the Fusion Event Utilizing a RAF Inhibitor

**Figure 5:**
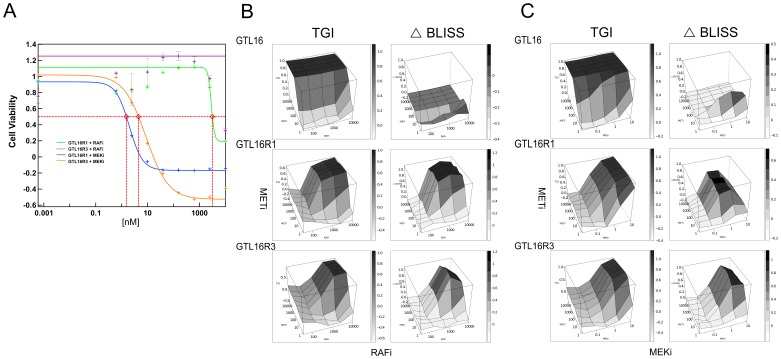
The c-Met inhibitor synergizes with either RAFi or MEKi. (A) Cell Viability of resistant GTL16R1 and GTL16R3 clones treated with RAFi or MEKi as single agents. Resistance of GTL16R1 and GTL16R3 clones to RAFi single agent treatment suggests that c-Met is involved in signaling independent of Raf. MEKi treatment inhibits cell viability as a single agent with IC50 values of 1.5 nM and 4.5 nM for GTL16R1 and GTL16R3, respectively. (B) METi and RAFi synergistically inhibit tumor growth of GTL16R1 and GTL16R3 resistant lines. Wildtype GTL16 (WT), resistant GTL16R1 and GTL16R3 clones were cultured with a combination of METi and RAFi at the indicated nM concentrations. Matrix grid represents various concentration combinations. Single agent activity can be seen for METi and RAFi on the respective edges of the plot. Tumor cell growth inhibition (TGI) or ΔBLISS independence is indicated on the Z-axis and shaded according to the value in the legend. TGI ranges from low (no effect on growth) to high (complete suppression of growth). ΔBLISS ranges from low (complete independence/additivity) to high (synergy). (C) METi and MEKi can synergize in GTL16R1 and GTL16R3. Wildtype GTL16 (WT), resistant GTL16R1 and GTL16R3 clones were cultured with a combination of METi and MEKi at the indicated nM concentrations. Matrix grid represents various concentration combinations. Single agent activity can be seen for METi and MEKi on the respective edges of the plot. Tumor cell growth inhibition (TGI) or ΔBLISS independence is indicated on the Z-axis and shaded according to the value in the legend. TGI ranges from low (no effect on growth) to high (complete suppression of growth). ΔBLISS ranges from low (complete independence/additivity) to high (synergy).

To test the functional significance of the BRAF fusion, we treated with METi, RAFi, or a combination of both in cell viability assays. Single agent treatment with RAFi did not alter growth of GTL16, GTL16R1 or GTL16R3 (Figure 5A and B, x-axis). However, GTL16R1 and GTL16R3 treated with METi in combination with RAFi demonstrated an effective tumor cell growth inhibition (TGI). To assess whether this combination was additive or synergistic, we calculated the ΔBLISS score (see methods). As shown in figure 5B, GTL16R1 and GTL16R3 both have a maximal ΔBLISS score of 1 indicating a synergistic effect. Maximal synergy occurs between >39 nM METi and >625 nM RAFi for GTL16R1 while GTL16R3 achieves max synergy >39 nM METi and >2.5 µM RAFi. Treating GTL16 with this same combination of METi and RAFi did not demonstrate additional growth inhibition compared with METi alone, consistent with oncogenic addiction to c-Met signaling.

### GTL16 and the Resistant Clones are Dependent upon MAPK Signaling

To further confirm that the BRAF fusion protein is indeed signaling through the MAPK pathway, we treated GTL16R1 and GTL16R3 with MEKi as a single agent or in combination with METi. Surprisingly, unlike RAFi, single agent MEKi was very potent and was able to inhibit tumor cell growth with an IC50 value of 1.5 and 4.5 nM for GTL16R1 and GTL16R3, respectively (Fig 5A). GTL16 cells were also inhibited by single agent MEKi with an IC50 value of 8.2 nM (Figure 5C, x-axis).

To determine if MEKi can synergize with METi, we tested with a sub-optimal dose of MEKi (ranging from 10 nM to 156 pM). Combination with 2.5 nM of MEKi yielded the best synergy as demonstrated by the ΔBLISS plot (Figure 5C).

### A Combination of METi and RAFi Increased Inhibition of ERK Phosphorylation in the Resistant Clones

**Figure 6:**
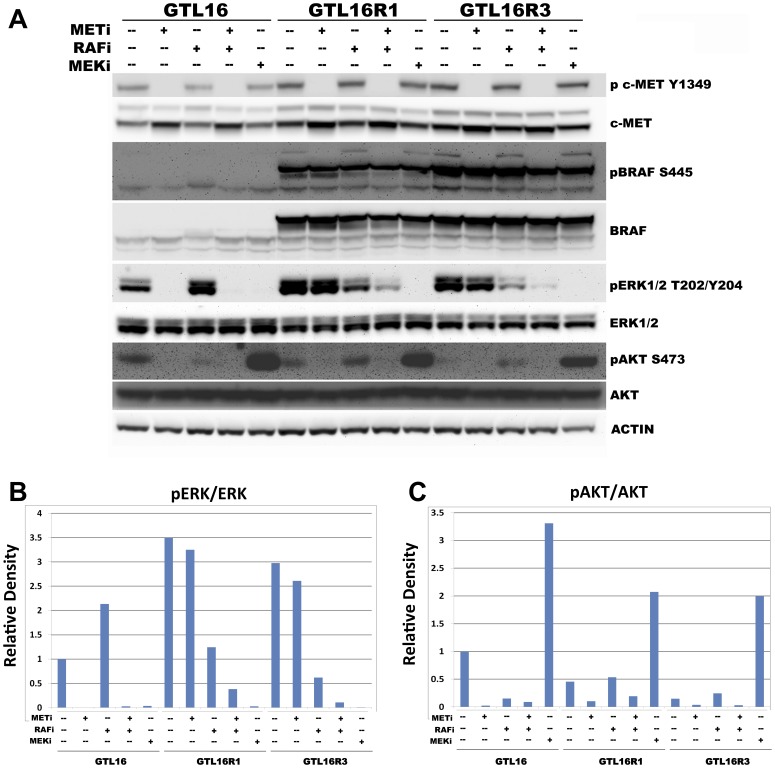
Higher ERK activity coincides with highly expressed SND1-BRAF fusion proteins in GTL16R1 and GTL16R3. (A) Western immunoblot of GTL16, GTL16R1 and GTL16R3 treated with inhibitors as single agent or combinations for 4 hr. (B) Densitometry measurement of the relative intensity of phospho ERK normalized to total ERK within each sample. (C) Densitometry measurement of the relative intensity of phospho AKT S473 normalized to total AKT within each sample.

To elucidate the mechanism of synergy, we examined modulation of signal transduction pathways by Western immunostaining total cell lysates from GTL16, GTL16R1 and GTL16R3 treated with 200 nM of METi, RAFi, or MEKi as single agents and in combination (Figure 6). Phosphorylation of the c-Met Y1349 docking site was consistently inhibited in GTL16, GTL16R1, and GTL16R3 by METi, but not by RAFi or MEKi alone (Figure 6A). This inhibition was achieved despite moderately increased levels of total c-Met protein upon METi treatment, suggesting that METi blocks c-Met activity in GTL16R1 and GTL16R3 with similar efficacy as GTL16 (Figure 6A).

In GTL16R1 and GTL16R3, SND1-BRAF was constitutively phosphorylated on BRAF S445 and levels did not respond significantly relative to total SND1-BRAF protein upon treatment with any inhibitor (Figure 6A). The Serine 445 residue has been reported to be constitutively phosphorylated in BRAF [43], and would not be expected to be affected by inhibitors of BRAF activity.

The c-Met tyrosine receptor kinase activates two major signal transduction cascades, the RAS-RAF-ERK MAP Kinase pathway and the PI3K/AKT pathway. We examined AKT activity via phosphorylation of S473 on AKT using Western immunoblot. Compared to GTL16, untreated levels of AKT pS473 were approximately two-fold and six-fold lower in GTL16R1 and GTL16R3, respectively (Figure 6A and C). Treatment with METi reduced AKT S473 phosphorylation to <10% in all cell lines. RAFi alone reduced AKT pS473 levels in GTL16, while it increased pS473 levels slightly in GTL16R1 and GTL16R3. A combination of METi and RAFi decreased AKT pS473 to levels comparable to METi alone. In contrast, treatment with MEKi induced an approximately three-fold increase of AKT pS473 in all cell types.

We also investigated ERK phosphorylation levels in response to compound treatments. The basal phosphorylated ERK levels in GTL16R1 and GTL16R3 were approximately 3 fold higher than GTL16. In response to METi, ERK phosphorylation was completely inhibited in GTL16, however, ERK phosphorylation only decreased slightly in GTL16R1 and GTL16R3 (Figure 6A and B).

Treatment with RAFi increased ERK phosphorylation two fold in GTL16 as RAF inhibitors can transactivate homo/heterodimers of RAF isoforms to increase MEK signaling [44], [45]. Despite the increased level of ERK phosphorylation, we did not observe an increase in cell proliferation. Although treatment of GTL16R1 and GTL16R3 with RAFi decreased ERK phosphorylation by approximately three fold, the level of phosphorylated ERK remained comparable to untreated GTL16 (Figure 6B). This could explain why RAFi alone did not inhibit cell proliferation of the resistant clones (Figure 5B) because the GTL16 cells are being driven by hyperactivation of c-Met signaling which activates MAPK through a RAF independent mechanism. Since METi completely blocks ERK phosphorylation in GTL16 cells, there are likely no activating Ras mutations or other activated downstream mediators of MAPK signaling. Only the treatment of GTL16R1 and GTL16R3 with a combination of METi and RAFi further inhibited ERK phosphorylation to 38% and 10.5% of untreated GTL16 (Figure 6B).

Since GTL16R1 and GTL16R3 seemed to shift signaling towards the MAPK pathway and away from the PI3K/AKT pathway, we tested whether the resistance was solely dependent upon the MAPK pathway using MEKi alone. Single agent MEKi was able to completely inhibit ERK phosphorylation in all cells. Consistent with BRAF activation, these data indicate that METi inhibition is bypassed downstream of c-Met and upstream of MEK.

## Discussion

In some cancers, dysregulated c-Met signaling results in oncogene addiction, a dependence on the pathway to maintain cancer cell growth and survival [46]. The *MET* locus is amplified or mutated in selected tumor types, and these patients are predicted to be responsive to treatment with c-Met inhibitors [8], [47]. Unfortunately, acquired resistance is a significant concern for single agent therapy based on precedence with agents like imatinib in CML and erlotinib in lung adenocarcinoma [48].

**Figure 7:**
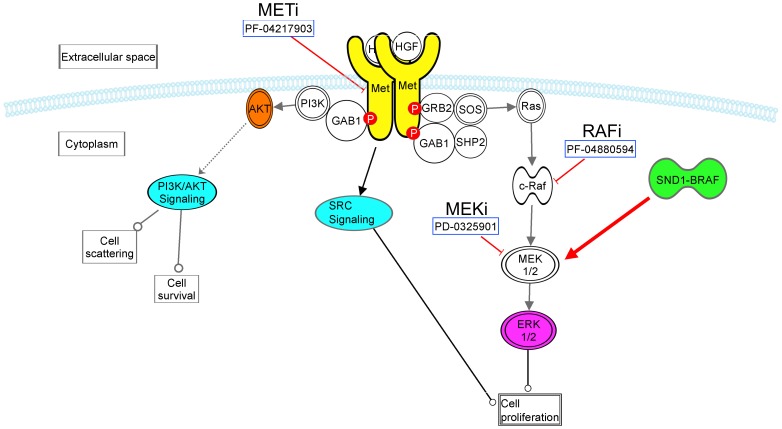
A model highlighting the resistance mechanism in GTL16 GTL16R1 and GTL16R3. Highly expressed SND1-BRAF fusion protein signals through the MEK/ERK pathway and short-circuits c-Met inhibition.

Here we describe a GTL16 cell line model of METi resistance. We identify an activated SND1-BRAF fusion protein which confers resistance to METi. BRAF promotes cell growth and proliferation by transducing signals from growth factor receptors as part of the MAP kinase pathway via MEK and ERK. Our findings are consistent with a model of resistance where the fusion activated BRAF can bypass upstream c-Met inhibition by hyperactivating the MAPK pathway in a c-Met independent manner. As summarized in figure 7, several signaling cascades are activated by the c-Met receptor including PI3K, SRC, and Ras signaling. By inhibiting tyrosine phosphorylation of critical c-Met residues used to recruit adapter proteins GRB2 and GAB1, METi inhibits the recruitment and activation of Ras and prevents activation of the MAPK pathway by the c-Met receptor. The SND1-BRAF fusion, however, circumvents METi by signaling downstream of the c-Met receptor to MEK. The resistance can be overcome by treating with MEKi, or a combination of METi and RAFi to target the SND1-BRAF fusion protein.

Like previously described BRAF fusion proteins, the specific role of the N-terminal partner seems limited. Despite containing an intriguing array of tandem Staphylococcal nuclease homologue domains which function to capture RNA targets, the SND1 portion of the fusion protein likely does not directly contribute to c-Met resistance because use of RAFi was able to restore sensitivity to METi. Rather, we think transcription of the gene fusion is driven by the SND1 promoter and SND1 replaces the N-terminal regulatory region of BRAF which normally regulates BRAF catalytic activity [49].

Importantly, the simultaneous combination treatment with both METi and RAFi is required to reduce the phosphorylation of ERK in GTL16R1 and GTL16R3 more than 50% compared to basal pERK levels in GTL16 (Figure 6B). The inability of RAFi to completely inhibit ERK phosphorylation and reduce cell proliferation as a single agent suggests that c-Met signaling activates MEK independent of RAF catalytic activity. In this model, both the c-Met and BRAF pathways would need to be blocked to reduce ERK phosphorylation, enough to inhibit cell proliferation. Additionally, as revealed by RPPA in figure 2, METi treatment decreases SRC activity, and may suggest that SRC dependent signaling is another pathway active in GTL16 cells. Bertolli et al. demonstrates that adding SRC inhibitors enhances inhibition of cell viability by c-Met inhibition [50].

The role of PI3K activation in our particular resistant model seems minor. Lower basal pAKT S473 levels in GTL16R1 and GTL16R3 suggest less dependence upon PI3K signaling. Additionally, while METi treatment suppresses pAKT S473, MEKi treatment dramatically increases pAKT S473, and yet still inhibits cell viability, suggesting that suppressing AKT phosphorylation is not a requirement for blocking cell proliferation (Figure 5A and C). Indeed, these results are consistent with experiments by Hoeflich et.al. where increases in AKT p473 were seen in response to MEKi in several cell lines. While increases in AKT/PI3K activity are associated with anti-apoptosis and enhanced cell survival, Hoeflich et.al. postulated that AKT/PI3K pathway activation in response to MAPK pathway inhibition is delayed and may be too late to maintain cell viability [51]. Hence, in our model, signaling through MEK seems sufficient for rescue from c-Met inhibition.

It is unclear if prolonged exposure of GTL16 cells to METi resulted in *de novo* SND1-BRAF fusion translocations, or if resistant clones arose from an enrichment of pre-existing cells with these translocations. The GTL16R1 and GTL16R3 are not identical clones despite possessing the same SND1-BRAF fusion protein product. RNA-Seq and exome sequencing data show clone- specific SNPs. Furthermore, the CNV segmentation pattern appears to be distinct in the 7q32–7q34 region. However, because the breakpoints are so similar, it is possible that GTL16R1 and GTL16R3 are subvariants of a common precursor cell that was present in the GTL16 tumor which has since diverged over time creating a subpopulation of SND1-BRAF cells in culture. Additionally, the SND1-BRAF fusion is highly amplified in GTL16R1 and GTL16R3, but is not detected via Next Generation Sequencing in GTL16. At this point we cannot rule out the possibility of a small pre-existing pool of SND1-BRAF expressing clones in GTL16.

The clinical significance of the SND1-BRAF fusion is unknown. A computational search of publically available CNV arrays representing more than 5000 cell lines and primary samples did not identify any co-occurring SND1-BRAF breakpoints consistent with the known fusion. It is likely that selective pressure from prolonged drug treatment is required to generate SND1-BRAF fusions in primary tumors.

The acquisition of additional oncogenic drivers has important implications for targeted therapy. The sequential treatment of tumors with targeted agents may allow sequential resistance to develop if inhibitors are applied individually. Conceivably, resistance mechanisms to BRAF inhibition like IGF-1R [52], COT [53], NRAS [54], or PDGFRbeta [54] could also provide alternate survival pathways in the context of c-Met inhibition. For example, EGFR signaling is one example of a resistance mechanism common to both c-Met inhibition [2] and BRAF inhibition [55]. Additionally, in a study of BRAF mutated Papillary Thyroid Carcinoma (PTC), c-Met expression was 3-fold higher in the BRAF aggressive PTC vs. the BRAF non-aggressive PTC [56]. Furthermore, c-Met amplification is a primary resistance mechanism to the BRAF inhibitor PLX4032 in patient derived melanoma cell lines [57]. Hence, c-Met and BRAF co-activation may increase tumor robustness and resistance to targeted therapy due to activation of multiple growth and survival pathways. Further studies are needed to understand the complementarity and essential elements of c-Met and BRAF inhibition.

## Supporting Information

Figure S1 Kinase Selectivity Screen of METi. (A) Kinase selectivity screen (KSS) performed at Upstate Biotechnology. Values in % inhibition of phosphorylation given 1 µM of METi compared to control. (B) KSS performed at University of Dundee (Division of Signal Transduction Therapy). Values in % inhibition of phosphorylation given 1 µM of METi compared to control. (C) Cell based dose response kinase inhibition of indicated kinases by METi.
(PDF)

Figure S2 Kinase Selectivity Screen of RAFi. (A) Kinase selectivity screen (KSS) performed at University of Dundee (Division of Signal Transduction Therapy). Values in % inhibition of phosphorylation given 1 µM of RAFi compared to control. (B) KSS performed at Invitrogen (Selectscreen Service) compared to control. Values in % inhibition of phosphorylation given 1 µM of RAFi. (C) Cell based dose response kinase inhibition of indicated kinases by RAFi.
(PDF)

Figure S3 Phosphorylation fold decreases upon PF-04217903 treatment of GTL16, GTL16R1 and GTL16R3 cells compared to vehicle. Values are taken from RPPA analysis of cells treated with 2.5 µM METi for 1 hr.
(PDF)

Figure S4 Amplification at regions 7q34 and 7q32 form a fusion transcript. (A) CNV data show breakpoints between exons 16–17 of SND1 and 8–9 of BRAF. (B) RNA-Seq paired end reads aligned to the putative fusion transcript show reads that overlap or span the SND/BRAF junction only in clone GTL16R3 but not GTL16.
(PDF)

Figure S5 (A) Table of raw coverage or normalized Reads/Base/Million (RBM) reads of RNA-Seq that align to full length or fusion regions of SND1 or BRAF. Fold is calculated relative to GTL16. Fusion regions are overrepresented in GTL16R1 and GTL16R3 relative to GTL16. (B) Graphic of the raw coverage and RBM at each base position in SND1 and BRAF shows the uneven distribution of coverage and high expression of the fusion transcript.
(PDF)
