# Correction: A Novel SND1-BRAF Fusion Confers Resistance to c-Met Inhibitor PF-04217903 in GTL16 Cells though MAPK Activation

**DOI:** 10.1371/annotation/d988dd75-bcd2-4859-b20b-487e1bf2513b

**Published:** 2012-08-20

**Authors:** Nathan V. Lee, Maruja E. Lira, Adam Pavlicek, Jingjing Ye, Dana Buckman, Shubha Bagrodia, Sreesha P. Srinivasa, Yongjun Zhao, Samuel Aparicio, Paul A. Rejto, James G. Christensen, Keith A. Ching

The word "through" is misspelled in the article title. The correct title is: A Novel SND1-BRAF Fusion Confers Resistance to c-Met Inhibitor PF-04217903 in GTL16 Cells through MAPK Activation. The correct citation is:

Lee NV, Lira ME, Pavlicek A, Ye J, Buckman D, et al. (2012) A Novel SND1-BRAF Fusion Confers Resistance to c-Met Inhibitor PF-04217903 in GTL16 Cells through MAPK Activation. PLoS ONE 7(6): e39653. doi:10.1371/journal.pone.0039653 

