# Supplementary material for: A Novel SND1-BRAF Fusion Confers Resistance to c-Met Inhibitor PF-04217903 in GTL16 Cells though MAPK Activation
Source: PLoS One. 2012 Jun 22;7(6):e39653. doi: 10.1371/journal.pone.0039653 (PMC3382171; doi:10.1371/journal.pone.0039653)
Supplement: Figure S1 — Kinase Selectivity Screen of METi. (A) Kinase selectivity screen (KSS) performed at Upstate Biotechnology. Values in % inhibition of phosphorylation given 1 µM of METi compared to control. (B) KSS performed at University of Dundee (Division of Signal Transduction Therapy). Values in % inhibition of phosphorylation given 1 µM of METi compared to control. (C) Cell based dose response kinase inhibition of indicated kinases by METi. (PDF) [file pone.0039653.s001.pdf]

A.

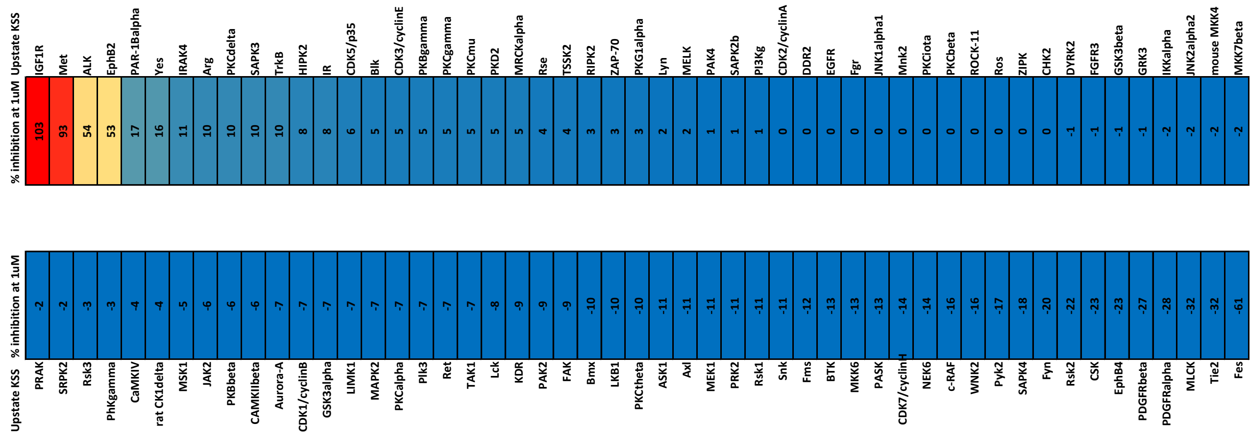

B.

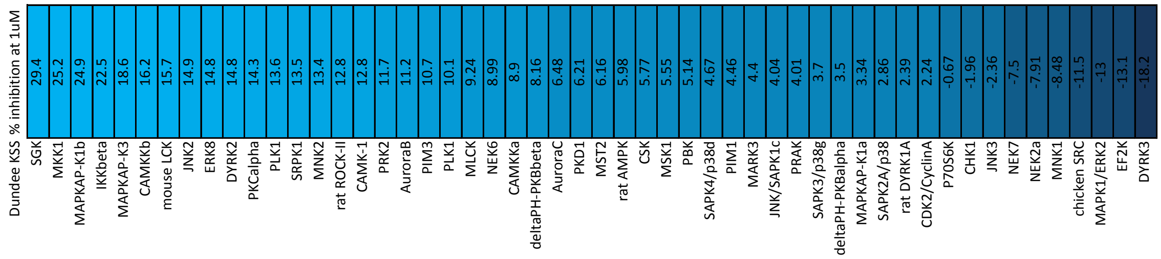

C.

Kinase	*IC50 (nM)	Selectivity Ratio
c-Met	6.7	---
RON	>10,000	>1,600X
IRK	>10,000	>1,600X
IGF1R	>10,000	>1,600X
ALK	>10,000	>1,600X

Cellular Selectivity on Selected Kinases

*the cellular kinase activities were measured using ELISA capture method
