# Supplementary material for: A Novel SND1-BRAF Fusion Confers Resistance to c-Met Inhibitor PF-04217903 in GTL16 Cells though MAPK Activation
Source: PLoS One. 2012 Jun 22;7(6):e39653. doi: 10.1371/journal.pone.0039653 (PMC3382171; doi:10.1371/journal.pone.0039653)
Supplement: Figure S2 — Kinase Selectivity Screen of RAFi. (A) Kinase selectivity screen (KSS) performed at University of Dundee (Division of Signal Transduction Therapy). Values in % inhibition of phosphorylation given 1 µM of RAFi compared to control. (B) KSS performed at Invitrogen (Selectscreen Service) compared to control. Values in % inhibition of phosphorylation given 1 µM of RAFi. (C) Cell based dose response kinase inhibition of indicated kinases by RAFi. (PDF) [file pone.0039653.s002.pdf]

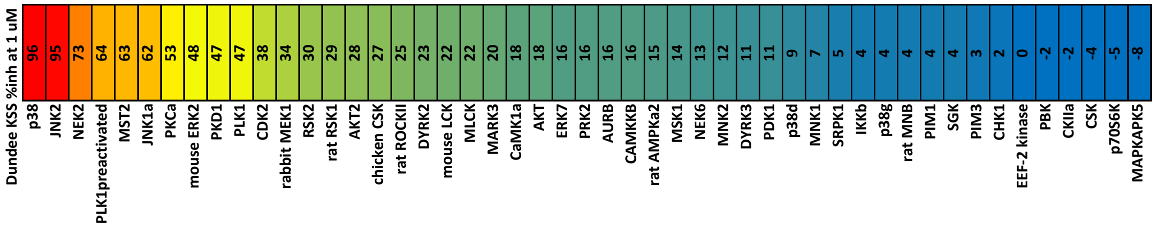

A.

B.

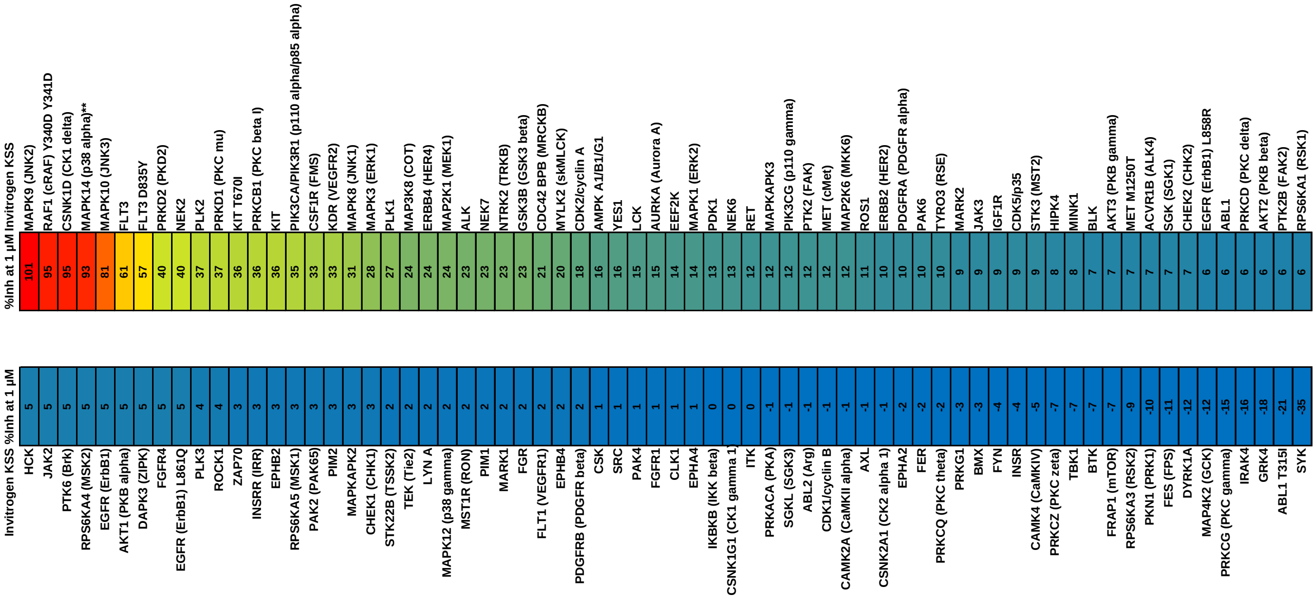

C.

Kinase	*IC50 (nM)	Selectivity Ratio
BRAF	0.19	---
BRAF V599E	0.13	0.68x
c-RAF	0.39	2x
CSNK1D	51.9	273x
JNK2	23	121x
JNK3	120	632x
P38	176	926x

Cellular Selectivity on Selected Kinases

*the cellular kinase activities were measured using ELISA capture method
