# Supplementary material for: A Novel SND1-BRAF Fusion Confers Resistance to c-Met Inhibitor PF-04217903 in GTL16 Cells though MAPK Activation
Source: PLoS One. 2012 Jun 22;7(6):e39653. doi: 10.1371/journal.pone.0039653 (PMC3382171; doi:10.1371/journal.pone.0039653)
Supplement: Figure S3 — Phosphorylation fold decreases upon PF-04217903 treatment of GTL16, GTL16R1 and GTL16R3 cells compared to vehicle. Values are taken from RPPA analysis of cells treated with 2.5 µM METi for 1 hr. (PDF) [file pone.0039653.s003.pdf]

	EGFR pY1068	EGFR pY1173	ERRB2 pY1248	ERBB3 pY1289	ERBB4 pY1284	MET pY1235	SRC pY416
GTL16 parental	6.60	4.39	3.02	2.27	2.34	4.30	6.97
GTL16 R1	6.79	3.88	2.76	1.78	1.48	4.42	5.47
GTL16 R3	3.09	2.75	1.63	2.00	1.86	3.47	3.15
