# Supplementary figures and images for: A Novel SND1-BRAF Fusion Confers Resistance to c-Met Inhibitor PF-04217903 in GTL16 Cells though MAPK Activation
Source: PLoS One. 2012 Jun 22;7(6):e39653. doi: 10.1371/journal.pone.0039653 (PMC3382171; doi:10.1371/journal.pone.0039653)

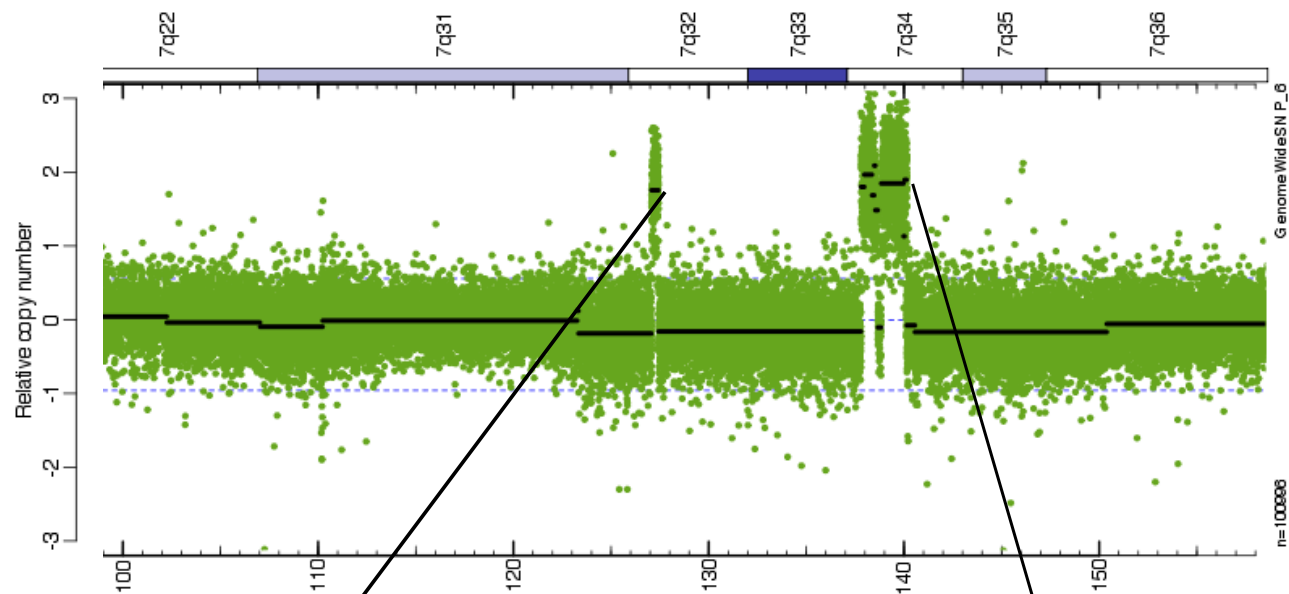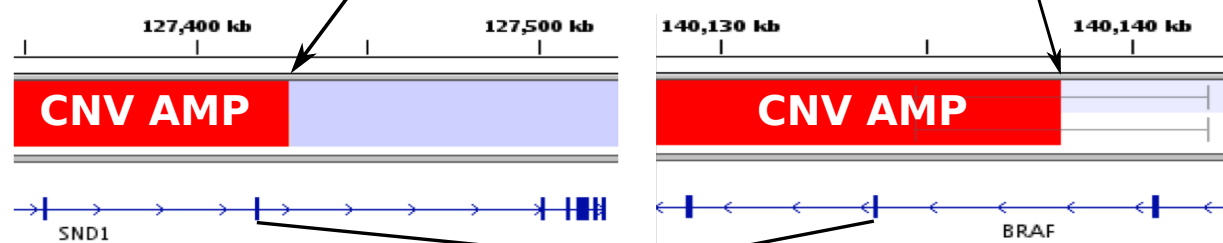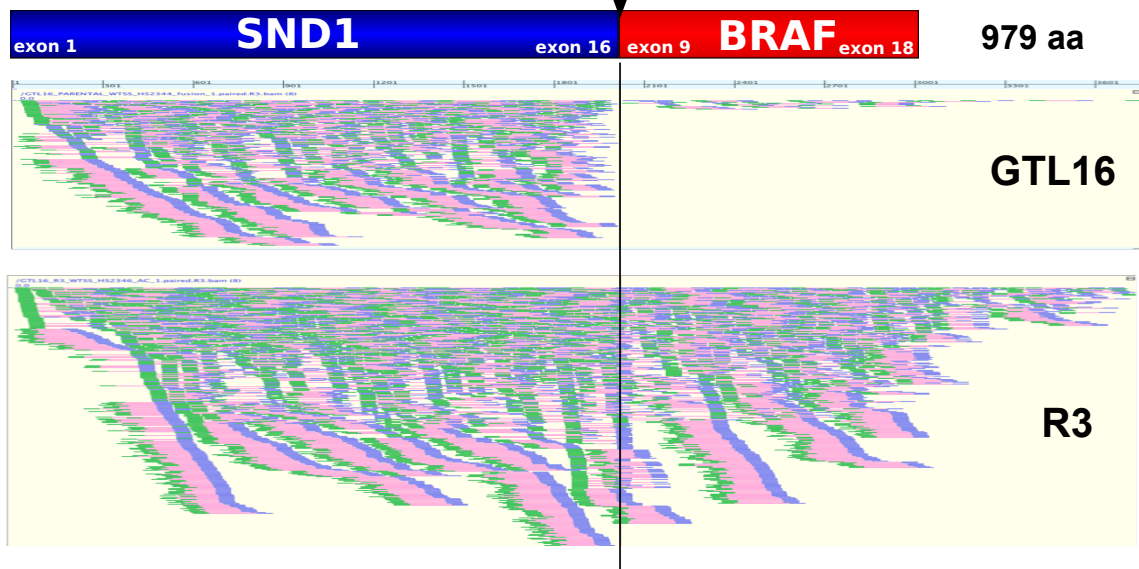

Supplement: Figure S4 — Amplification at regions 7q34 and 7q32 form a fusion transcript. (A) CNV data show breakpoints between exons 16–17 of SND1 and 8–9 of BRAF. (B) RNA-Seq paired end reads aligned to the putative fusion transcript show reads that overlap or span the SND/BRAF junction only in clone GTL16R3 but not GTL16. (PDF) [file pone.0039653.s004.pdf]
