# Supplementary material for: A Novel SND1-BRAF Fusion Confers Resistance to c-Met Inhibitor PF-04217903 in GTL16 Cells though MAPK Activation
Source: PLoS One. 2012 Jun 22;7(6):e39653. doi: 10.1371/journal.pone.0039653 (PMC3382171; doi:10.1371/journal.pone.0039653)

	GTL16	R1	fold	R3	fold
SND1 coverage	124.840	267.640	2.144	180.790	1.448
SND1 RBM	0.517	1.050	2.031	0.863	1.669
SND1 1-2006 coverage	150.140	380.150	2.532	265.050	1.765
SND1 1-2006 RBM	0.622	1.492	2.399	1.265	2.034
SND1 2007-3522 coverage	91.280	118.760	1.301	66.570	0.729
SND1 2007-3522 RBM	0.378	0.466	1.233	0.318	0.841
BRAF coverage	10.060	109.640	10.899	80.480	8.000
BRAF RBM	0.042	0.430	10.238	0.384	9.143
BRAF 1-1201 coverage	13.760	13.200	0.959	11.650	0.847
BRAF 1-1201 RBM	0.057	0.052	0.912	0.056	0.982
BRAF 1202-2947 coverage	7.130	175.840	24.662	122.630	17.199
BRAF 1202-2947 RBM	0.030	0.690	23.000	0.585	19.500

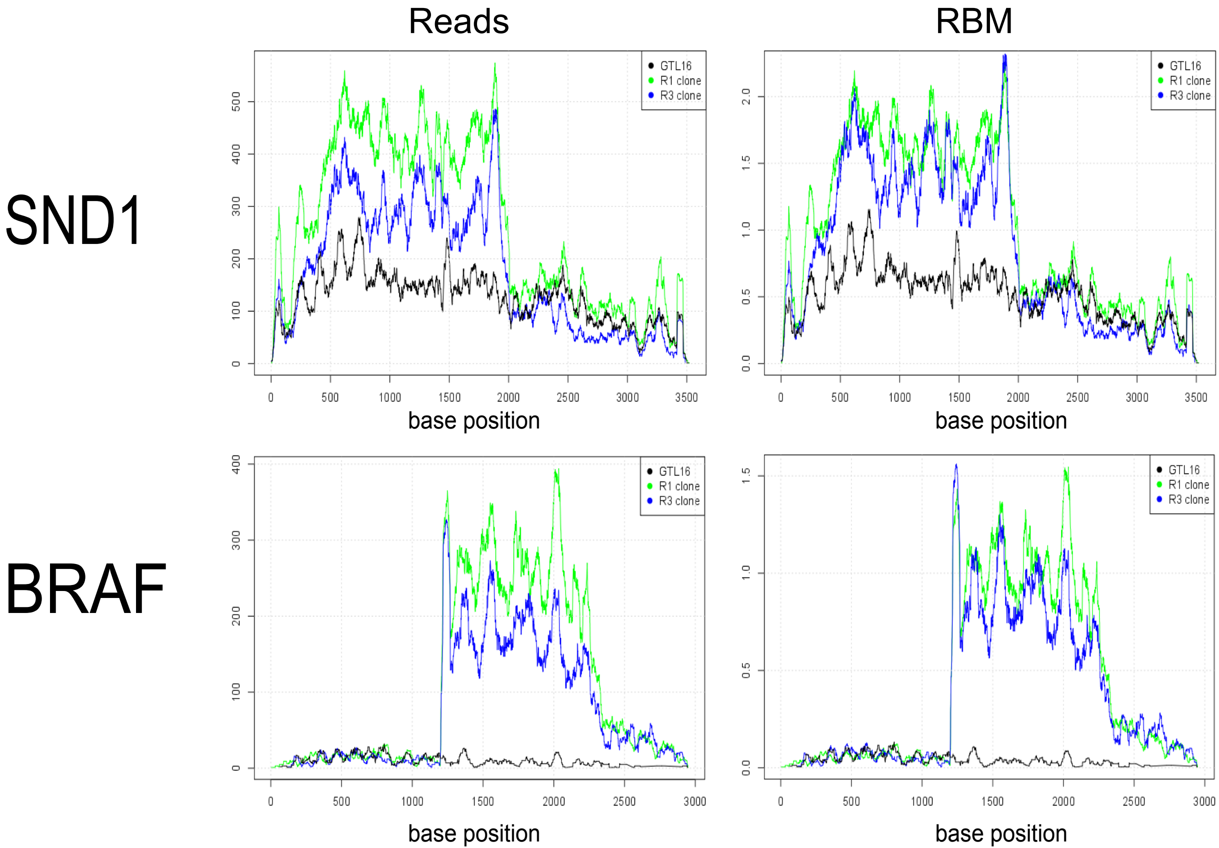

Supplement: Figure S5 — (A) Table of raw coverage or normalized Reads/Base/Million (RBM) reads of RNA-Seq that align to full length or fusion regions of SND1 or BRAF. Fold is calculated relative to GTL16. Fusion regions are overrepresented in GTL16R1 and GTL16R3 relative to GTL16. (B) Graphic of the raw coverage and RBM at each base position in SND1 and BRAF shows the uneven distribution of coverage and high expression of the fusion transcript. (PDF) [file pone.0039653.s005.pdf]
